# Sleep, Lifestyle, and Socioeconomic Markers of Mental Aging and Well-Being: Lessons From England, China, and Japan

**DOI:** 10.1093/geroni/igab046.459

**Published:** 2021-12-17

**Authors:** Andrew Steptoe

## Abstract

Healthy ageing has become a popular topic worldwide. We investigated the role of sleep, leisure activities, and socioeconomic inequalities in relation to cognitive decline, wellbeing, and quality of life in data from the English Longitudinal Study of Ageing (ELSA), Chinese Health and Retirement Longitudinal Study (CHARLS), and Japanese Study of Aging and Retirement (JSTAR), national representative samples of England, China and Japan, respectively. We found an inverted U-shaped association between sleep quality and memory in English adults and a positive dose-response association in Chinese older adults (Brocklebank). In another examination, we found that younger English individuals playing games had lower quality-of-life than older participants who game, and this association is more pronounced for widowed individuals than others (Almeida-Meza). Cognitive impairment and dementia represent significant challenges worldwide. In a cross-country investigation, we found that the prevalence of MCI was twice as great in England compared with Japan, but that the two nations differ slightly across socioeconomic correlates (Gireesh). In another cross-country comparison between England and China, we found that the rate of memory change appeared socioeconomically patterned, primarily by education and area-based characteristics (urban vs. rural), with a more substantial impact on rural China inequalities compared to England (Cadar). Our results indicate more robust educational and geographical disparities in China and increased occupational impact among English and Japanese participants. Our findings highlight the imperative need for policy interventions and tailored strategies to protect those particularly disadvantaged in England and China.

